# Feasibility and clinical efficacy of minimally invasive techniques in the treatment of type Ⅱ/Ⅲ brucellar spondylitis

**DOI:** 10.3389/fsurg.2026.1862072

**Published:** 2026-08-13

**Authors:** Xinming Yang, Yakun Du, Ye Tian, Yao Yao, Lixing Chen, NingKui Niu

**Affiliations:** 1 Department of Orthopedics, First Affiliated Hospital of Hebei North University, Zhangjiakou, China; 2 Department of Neuroelectrophysiology, Hebei Children's Hospital, Shijiazhuang, China; 3 General Hospital of Ningxia Medical University, Yinchuan, China

**Keywords:** brucellar spondylitis, clinical efficacy, CT-guided abscess drainage, debridement, intervention heterogeneity, minimally invasive spine surgery, percutaneous spine endoscopy, retrospective bias

## Abstract

**Objective:**

To explore the clinical feasibility and perioperative superiority of combined minimally invasive techniques in treating type II/III Brucellar Spondylitis (BS), clarify the stratified surgical indications for different minimally invasive procedures and key technical points, provide evidence-based medical evidence for selecting appropriate surgical methods in the clinical treatment of type II/III BS, and provide a reference for formulating clinical norms for minimally invasive techniques in treating BS, thereby promoting the standardized and popularized application of minimally invasive spinal surgery techniques in the treatment of BS. Limitations of the retrospective single-center design and mixed minimally invasive intervention heterogeneity are fully discussed to avoid overgeneralized inference.

**Methods:**

A retrospective controlled study was conducted with stratified subgroup description of different minimally invasive procedures and clear preoperative allocation criteria for surgical approaches to reduce selection bias. Patients diagnosed with type II/III BS and undergoing surgical treatment in our hospital from January 2020 to March 2025 were selected as the research subjects. They were divided into the minimally invasive group and the traditional open surgery group according to standardized preoperative surgical allocation criteria based on abscess scale, vertebral destruction range, spinal instability degree and spinal canal occupation ratio. The minimally invasive group integrated three types of distinct minimally invasive techniques including unilateral biportal endoscopy (UBE), percutaneous spine endoscopy, and CT-guided lesion drainage combined with minimally invasive percutaneous internal fixation; baseline lesion characteristics of patients receiving different minimally invasive modalities were separately described to reflect intervention heterogeneity. The open group underwent conventional anterior or posterior open debridement, bone graft fusion, and pedicle screw internal fixation. Through postoperative follow-up, the clinical baseline data, perioperative core indicators, pain visual analogue scale (VAS) score, Oswestry Disability Index (ODI), erythrocyte sedimentation rate (ESR), C-reactive protein (CRP) inflammatory indicators, ASIA neurological function grading, radiological bone fusion rate, postoperative complication occurrence rate and disease recurrence rate of the two groups of patients were systematically compared. Effect size calculation and minimal clinically important difference (MCID) analysis were supplemented for all continuous outcome indicators to distinguish statistical difference from clinical meaningful difference.

**Results:**

A total of 72 patients met the inclusion criteria, among which 38 were in the minimally invasive group and 34 were in the open surgery group. There were no statistically significant differences in clinical data such as gender, age, type of BS, affected vertebrae segments, presence of paravertebral abscess, and ASIA classification of neurological function between the two groups (*P* > 0.05); supplementary stratified comparison of abscess size, vertebral destruction severity and spinal canal occupation ratio between groups was performed to further verify intergroup clinical comparability, indicating good comparability. The minimally invasive group was significantly superior to the open group in perioperative indicators including operation time, intraoperative blood loss, surgical incision length, postoperative bed rest time, and hospital stay (*P* < 0.05), with medium-to-large effect sizes confirming clinical meaningful gaps. At 1 week, 1 month, 3 months, 6 months, and 12 months postoperatively, the reductions in VAS score, ODI, ESR, and CRP in the minimally invasive group were significantly greater than those in the open group (*P* < 0.05), and MCID thresholds were reached in most time points, representing clinically meaningful pain relief and inflammatory improvement. The excellent and good rate of neurological function recovery was 90.9% in the minimally invasive group and 75.0% in the open group, with no significant difference between the two groups (*P* > 0.05). The bone fusion rate was 92.1% in the minimally invasive group and 88.2% in the open group, showing comparable spinal fusion effects (*P* > 0.05). The postoperative complication rate was 21.05% in the minimally invasive group, significantly lower than 76.47% in the open group (*P* < 0.05), with large effect size supporting clinical safety advantages. At 12-month follow-up, the disease recurrence rate was 2.6% in the minimally invasive group and 8.8% in the open group, with no significant difference between the two groups (*P* > 0.05).

**Conclusion:**

Minimally invasive spine surgery techniques are safe and feasible in the treatment of Type Ⅱ/Ⅲ BS, achieving thorough debridement, reliable infection control reflected by reduced inflammatory markers, and sufficient nerve decompression. They are comparable to conventional open surgery in neurological recovery, clinical efficacy and spinal fusion outcomes, while possessing significant perioperative advantages such as less surgical trauma, less intraoperative bleeding, faster postoperative recovery, and lower complication rate. Considering the retrospective design, mixed minimally invasive intervention heterogeneity and lack of long-term follow-up beyond 12 months, minimally invasive techniques may serve as a preferred alternative surgical option for appropriately selected Type Ⅱ/Ⅲ BS patients who meet strict minimally invasive surgical indications, rather than a universal first-line scheme for all Type Ⅱ/Ⅲ BS cases.

## 1 Introduction

Brucellosis is a global zoonotic infectious disease caused by Gram-negative facultative intracellular bacteria of the genus Brucella, characterized by high infectivity, extensive organ involvement, high chronicity rate, and frequent recurrence (1). In recent years, the global incidence of human brucellosis has continued to rise, making it a public health issue seriously threatening public health and livestock industry development (2). The spine is the most common site for bone and joint brucellosis. When Brucella bacteria invade the spine, causing intervertebral discitis or vertebral inflammation, and affecting the adjacent soft tissues and muscles, it is called brucellar spondylitis (BS), accounting for 2% - 53% of patients with brucellosis. (3–5).

The core pathogenesis of BS is the colonization and proliferation of Brucella in vertebral bodies and intervertebral discs, inducing local inflammatory responses, which further lead to bone destruction, paraspinal abscess, spinal stenosis, and in severe cases, nerve/spinal cord compression and spinal instability. The lesion occurs most frequently in the lumbar spine, followed by the thoracolumbar segment; cervical and thoracic spine involvement is relatively rare (6, 7). Due to its insidious onset and non-specific clinical manifestations, BS mainly presents with persistent low back pain, low-grade fever, night sweats, fatigue, and weight loss in the early stage, which is easily confused with spinal tuberculosis, leading to a high clinical misdiagnosis rate (8, 9).

At present, there is no uniformity in the classification of BS in domestic and foreign literature. The existing classification methods are not conducive to clinical treatment and post-treatment imaging follow-up (10–12). Therefore, this study combines literature, clinical practicability, imaging features and disease severity to classify BS into three types: Type Ⅰ is simple discitis, only showing inflammatory edema of the intervertebral disc with mild vertebral bone destruction and no abscess or spinal instability; Type Ⅱ is vertebral bone and/or intervertebral disc destruction type, accompanied by localized paraspinal abscess, and some patients may have mild nerve root irritation without obvious spinal instability; Type Ⅲ is severe lesion type, presenting extensive bone destruction, giant paraspinal or psoas abscess, spinal malalignment and instability, with obvious spinal canal occupation and nerve/spinal cord compression symptoms, representing the key and difficult point in clinical treatment.

The core treatment strategy of BS is a combination of anti-infective therapy and surgical intervention, with anti-infective therapy as the basis throughout the course (13, 14). Most patients with Type Ⅰ BS achieve satisfactory outcomes with standard anti-infective therapy. For patients with Type Ⅱ and Ⅲ BS, due to extensive lesions, large abscess volume, severe intervertebral disc or bone destruction, and poor spinal stability, medication alone cannot completely eliminate lesions, control infection, or relieve nerve compression, so surgical intervention is required on the basis of anti-infective therapy (15). Conventional open surgery allows sufficient debridement and spinal stability reconstruction, but has disadvantages such as large incision trauma, extensive muscle dissection, massive intraoperative bleeding, severe postoperative pain, long rehabilitation period, and high complication rate (16, 17). Most BS patients are accompanied by malnutrition and hypoimmunity, resulting in poor tolerance to open surgery and further increased surgical risks (18).

With the advancement of spinal surgical instruments and techniques, minimally invasive spine surgery has become the development trend of spinal surgery due to its advantages of less trauma, less bleeding, rapid postoperative recovery, and low complications (19). Techniques such as unilateral biportal endoscopy (UBE), percutaneous spine endoscopy, percutaneous pedicle screw fixation, and CT-guided puncture drainage have been gradually applied in clinical practice. Notably, these three minimally invasive modalities differ substantially in surgical trauma, operative range, decompression capacity and applicable lesion spectrum, and pooling them into a unified observation group inevitably introduces intervention heterogeneity, which is a core methodological limitation of this study and will be discussed in detail in the Limitations section. Among them, UBE provides a clear visual field and sufficient operating space, enabling debridement, nerve decompression, and bone graft fusion, showing significant advantages in the treatment of infectious spondylitis. Existing studies have shown that minimally invasive techniques can achieve comparable efficacy to open surgery in the treatment of spinal tuberculosis and pyogenic spondylitis with less trauma (20, 21). In the treatment of BS, minimally invasive techniques can complete lesion debridement, decompression, and stability reconstruction under direct endoscopy, making them more suitable for immunocompromised patients with infections (3).

Current studies on minimally invasive techniques for BS are mostly small-sample, single-center retrospective analyses. Large-sample, long-term follow-up controlled studies on moderate to severe Type Ⅱ and Ⅲ BS are insufficient. Its feasibility, safety, infection control efficacy, and long-term fusion outcomes remain controversial, and unified standards for surgical indications, key techniques, and postoperative management have not been established, thus restricting clinical promotion (22, 23). In addition, existing retrospective comparative studies lack clear preoperative surgical allocation criteria, fail to stratify different minimally invasive procedures, rarely supplement effect size analysis to judge clinical significance, and ignore quality of life evaluation indicators, which this study attempts to partially compensate for while acknowledging inherent research defects.

Based on the current research status, the objective of this study is to explore the clinical feasibility and perioperative superiority of combined minimally invasive techniques in treating type II/III BS, clarify stratified surgical indications for different minimally invasive modalities and key technical points, provide evidence-based medical evidence for choosing appropriate surgical methods in the clinical treatment of type II/III BS, and also provide references for formulating clinical norms for minimally invasive treatment of BS, thereby promoting the standardized and popularized application of minimally invasive spinal surgery techniques in the treatment of BS. Meanwhile, multiple methodological limitations including mixed minimally invasive intervention types, potential selection bias of retrospective grouping, lack of long-term follow-up and quality-of-life indicators are fully elaborated to avoid overstated clinical conclusions.

## 2 Materials and methods

### 2.1 Research subjects and contents

The clinical data of 72 patients with type II/III BS who were confirmed through clinical symptoms, laboratory tests, and imaging examinations and underwent surgical treatment in our hospital from January 2020 to March 2025 were selected for retrospective analysis. The differences in perioperative indicators, pain relief, functional recovery, infection control, nerve function recovery, spinal fusion, complications, and recurrence rates between minimally invasive spinal surgery and traditional open surgery were systematically compared. Supplementary analyses including effect size calculation and MCID judgment were added for continuous outcome indicators; stratified descriptive statistics of lesion characteristics (abscess diameter, vertebral destruction scope, spinal canal occupation ratio) were supplemented for patients receiving three different minimally invasive procedures to describe intervention heterogeneity. This study was approved by the Medical Ethics Committee of Hebei North University First Affiliated Hospital (Ethics Approval No. 2020-10). Informed consent was waived due to the retrospective nature of the study.

#### 2.1.1 Inclusion criteria

1. Meeting the diagnostic criteria for brucellosis in the Guidelines for the Diagnosis and Treatment of Brucellosis (2023 Edition) (24), i.e., standard tube agglutination test titer≥1:100++, or positive blood/lesion tissue bacterial culture;
2. Definitely diagnosed with Type Ⅱ or Type Ⅲ BS by lumbothoracic MRI plain scan plus enhancement and CT three-dimensional reconstruction, conforming to the corresponding imaging and clinical classification criteria;
3. No significant improvement or even aggravation of clinical symptoms after receiving regular multi-drug combination anti-infective therapy for≥4 weeks preoperatively, or disease progression;
4. With clear surgical indications, including formation of paravertebral/lumbar muscle abscesses, significant bone destruction of the vertebrae, abnormal or unstable spinal physiological curvature, or nerve root or spinal cord compression resulting in neurological dysfunction (grade C or D);
5. Completing surgical treatment with postoperative follow-up ≥12 months and complete follow-up data of laboratory and imaging indicators;
6. Complete preoperative imaging measurement data including maximum abscess diameter, vertebral destruction range, spinal canal occupation ratio for intergroup balance verification.

### 2.2 Grouping method

According to the surgical treatment methods received by the patients, they were divided into the minimally invasive group and the open group. Unified preoperative surgical allocation criteria were formulated to reduce selection bias, with clear stratification rules for surgical scheme selection as follows:

1. Minimally invasive surgery eligibility standard: single-segment lesion, abscess maximum diameter ≤5 cm, spinal canal occupation <50%, mild spinal instability or stable spine, no multi-vertebral collapse or severe spinal deformity;
2. Open surgery eligibility standard: multi-segment vertebral destruction, abscess maximum diameter >5 cm with extensive fascial diffusion, spinal canal occupation ≥50%, severe spinal collapse/deformity, previous spinal surgery history with severe perispinal adhesion;
3. For borderline cases meeting partial above standards, surgical scheme was jointly determined by two senior spinal surgeons based on comprehensive evaluation of patient age, underlying diseases, immune status and surgical tolerance.

Unified preoperative screening and inclusion criteria were applied to all enrolled patients before grouping; there were no differential inclusion thresholds set for the two surgical groups. All subjects met the identical Type Ⅱ/Ⅲ brucellar spondylitis diagnostic standards, preoperative anti-infective treatment course, and surgical indication criteria listed in 2.1 Inclusion Criteria. The only difference in patient selection between groups lies in intraoperative surgical strategy allocation based on standardized preoperative lesion evaluation rather than arbitrary admission screening thresholds.

The minimally invasive group adopted three differentiated minimally invasive spinal surgical techniques with clear applicable lesion stratification:

1. CT-guided lesion drainage combined with percutaneous endoscopic debridement: suitable for type II BS, disc destruction with a large paravertebral localized abscess and spinal stability.
2. Unilateral biportal endoscopy (UBE) debridement and decompression: suitable for type II BS with moderate bone destruction, mild spinal canal compression, but without severe instability.
3. UBE combined with percutaneous pedicle screw fixation: suitable for type III BS, Severe destruction of intervertebral discs/bones, multiple abscesses in the paravertebral area/buttock wall, spinal instability, accompanied by significant spinal canal stenosis and compression of nerves/spinal cord.

Baseline lesion characteristics of patients receiving the three above minimally invasive sub-procedures were separately counted in supplementary descriptive data to reflect intervention heterogeneity.

The open group was treated with traditional anterior or posterior open lesion debridement, bone graft fusion, and pedicle screw internal fixation for severe complex lesions that failed minimally invasive eligibility standards.

### 2.3 Preoperative preparation

All patients underwent comprehensive preoperative examinations and preparations to ensure safe surgery, as follows.

1. Laboratory examinations Routine laboratory tests including blood routine, urine routine, stool routine, liver and kidney function, electrolytes, coagulation function, ESR, CRP, myocardial enzymes, blood glucose, blood albumin, and blood lipid were performed to evaluate systemic nutritional status, organ function, coagulation function, and surgical tolerance. Brucella standard tube agglutination test (SAT) was conducted, and blood culture or lesion puncture tissue culture was performed in some patients to confirm the diagnosis of brucellosis. No postoperative lesion tissue bacterial clearance culture data were collected in this study; ESR and CRP were used as indirect surrogate markers to reflect infection control status, which cannot fully distinguish surgical traumatic inflammatory response from residual active infection.
2. Imaging examinations All patients underwent full-spine MRI plain scan plus enhancement to determine the lesion site, vertebral bone destruction degree, intervertebral disc involvement, location and size of paraspinal abscess, spinal canal occupation, and nerve/spinal cord compression. CT three-dimensional reconstruction of the lesion segment was performed to clearly show the details of vertebral bone destruction, pedicle morphology, and spinal bony structure, providing imaging evidence for surgical planning and operation. Quantitative imaging indicators including maximum abscess diameter, vertebral bone destruction area, spinal canal occupation percentage were measured uniformly for each patient to conduct intergroup balance comparison.
3. Organ function evaluation ECG, cardiac ultrasound, and chest CT were performed to evaluate cardiac and pulmonary function. For patients >60 years old or with underlying diseases such as diabetes and hypertension, further examinations were conducted to actively control underlying diseases and bring blood pressure and blood glucose within the surgical tolerance range.
4. Preoperative anti-infective therapy All patients received standard multi-drug combination anti-infective therapy preoperatively. The first-line regimen was doxycycline (100 mg, orally, twice daily) plus rifampicin (450–600 mg, orally, once daily) plus sulfamethoxazole (2 tablets, orally, twice daily). For patients with large abscesses and significantly elevated inflammatory indicators, a third-generation cephalosporin (such as ceftriaxone sodium, 2.0 g, intravenous infusion, once daily) or quinolone antibiotics (such as levofloxacin, 0.5 g, intravenous infusion, once daily) should be used in combination with the first-line regimen. The anti-infective course lasted at least 2 weeks (8, 9). Surgery was performed electively after body temperature returned to normal, systemic toxic symptoms relieved, and inflammatory indicators such as ESR and CRP decreased.
5. Other preoperative preparations Preoperative education was provided to inform patients of the surgical purpose, approach, postoperative precautions, and rehabilitation training methods, relieving anxiety and fear and improving cooperation. Skin and intestinal preparation were completed 1 day before surgery; fasting for 8 h and water deprivation for 4 h preoperatively. Enteral or parenteral nutritional support was given to patients with malnutrition to improve nutritional status and immunity.

### 2.4 Surgical methods

All the surgeries were performed by the same experienced spinal surgery team from our orthopedic department. Vital signs including body temperature, heart rate, blood pressure, blood oxygen saturation, and ECG were routinely monitored intraoperatively to ensure the safety and uniformity of the surgery.

#### 2.4.1 Minimally invasive group

The minimally invasive group was treated with individualized UBE-based minimally invasive techniques combined with percutaneous spine endoscopy, CT-guided lesion drainage, and minimally invasive percutaneous internal fixation according to unified preoperative lesion stratification standards (abscess size, bone destruction degree, spinal stability, spinal canal occupation). The key steps were as follows.

1. **Postural positioning and marking** localization Prone position; C-arm x-ray machine was used for anteroposterior and lateral fluoroscopy to locate the diseased vertebra, mark the pedicle projection, surgical access, and puncture point.
2. **Establishment of working channels** At the marked surgical incision site, two skin small incisions approximately 4 cm apart were made based on the size of the instruments used. Observation channels were established for inserting the UBE endoscope, and working channels were inserted for various minimally invasive operation instruments.
3. **Endoscopic debridement** Under direct endoscopy, the lesion was clearly identified; pus, inflammatory granulation tissue, and sequestrum were gradually removed with minimally invasive instruments until normal vertebral bone and intervertebral disc tissue were exposed. For patients with large paraspinal abscess, CT-guided percutaneous puncture drainage was performed first to aspirate part of the pus before endoscopic debridement, improving debridement efficiency.
4. **Spinal canal decompression** For patients with spinal canal occupation and nerve/spinal cord compression, hyperplastic osteophytes, calcified ligaments, and lesion tissues protruding into the canal were carefully removed under endoscopy using a drill or osteotome to fully decompress the dural sac and nerve roots. Intraoperative neuroelectrophysiological monitoring was used to avoid nerve injury.
5. **Minimally invasive percutaneous internal fixation and bone graft fusion** For patients with moderate to severe spinal instability and severe destruction of intervertebral discs or vertebral bodies, percutaneous pedicle screws were inserted into the adjacent vertebrae under C-arm guidance, and connecting rods were placed to reconstruct spinal stability. Autologous bone or artificial bone was implanted into the bone defect after debridement to promote interbody fusion.
6. Irrigation and drainage After debridement, decompression, and internal fixation, the surgical area was repeatedly irrigated with large amounts of normal saline to remove residual lesions and blood clots. One or two drainage tubes were indwelt and fixed, and the incision was closed.

For Type Ⅱ BS patients with deep, small abscess and no obvious spinal instability, only CT-guided puncture drainage combined with percutaneous endoscopic debridement was performed without internal fixation to reduce surgical trauma.

Descriptive statistics of case distribution for three minimally invasive sub-techniques were supplemented in results section to display intervention composition and heterogeneity.

#### 2.4.2 Open group

The open group underwent conventional anterior or posterior open surgery according to unified preoperative lesion stratification standards (lesion site, abscess scale, spinal destruction severity). Retroperitoneal anterior approach was used for lower lumbar and sacral lesions, posterior midline approach for upper lumbar and thoracolumbar lesions, and combined anterior-posterior approach for some severe cases. Key steps were as follows.

1. Positioning and localization Prone position for posterior surgery and supine position for anterior surgery; C-arm fluoroscopy was used to locate the diseased vertebra and mark the incision.
2. Surgical incision and exposure Posterior surgery used posterior midline incision; skin, subcutaneous tissue, and lumbodorsal fascia were incised layer by layer, and paraspinal muscles were dissected along both sides of the spinous process to expose the lamina, pedicle, and transverse process. Anterior surgery used a paramedian or transverse abdominal incision; skin and subcutaneous tissue were incised, the retroperitoneal space was opened, peritoneum and abdominal organs were freed to expose the anterior vertebral body, and segmental vessels were ligated to fully expose the lesion.
3. Open debridement Under direct vision, destructive bone, sequestrum, and inflammatory intervertebral disc tissue were removed with osteotomes, curettes, and nucleus pulposus forceps; the paraspinal abscess was aspirated. The abscess wall tightly adhered to surrounding tissues was completely resected to avoid residue.
4. Spinal canal decompression and spinal fixation For patients with nerve/spinal cord compression, laminectomy and spinal canal enlargement were performed in posterior surgery to decompress the dural sac and nerve roots; subtotal vertebrectomy was performed in anterior surgery to relieve anterior spinal canal compression. Pedicle screw system was used for internal fixation and stability reconstruction. Autologous iliac bone or artificial bone was implanted into the bone defect for fusion.
5. Irrigation and drainage The surgical area was repeatedly irrigated with normal saline; drainage tubes were indwelt, and muscles, fascia, subcutaneous tissue, and skin were sutured layer by layer.

### 2.5 Postoperative management

Both groups received standardized postoperative management and rehabilitation.

1. General nursing Patients were placed supine without pillow for 6 h after returning to the ward; vital signs, consciousness, limb sensation, motor function, and incision bleeding/exudation were closely monitored.
2. Anti-infective therapy The same multi-drug anti-infective regimen as preoperatively was continued postoperatively, adjusted according to recovery and drug sensitivity test results. The total anti-infective course was≥12 weeks (8, 9). Inflammatory indicators were regularly reviewed to evaluate infection control; intravenous antibiotics were switched to oral sequential therapy after indicators normalized and symptoms relieved. Limitations of ESR/CRP as infection evaluation markers: these indicators rise after major surgical trauma independent of active infection, and lack bacterial culture verification of lesion clearance, so infection control conclusions are indirect auxiliary evidence.
3. Drainage tube management Drainage tubes were kept unobstructed and recorded. The tube was removed when 24-hour drainage volume <20 mL and no hematoma/effusion was confirmed by imaging.
4. Nutritional support and symptomatic treatment Enteral or parenteral nutritional support was given; high-protein, high-vitamin, high-calorie digestible food was encouraged. Correct anemia and hypoproteinemia. Non-steroidal anti-inflammatory analgesics or opioids were used for pain relief. Underlying diseases such as hypertension and diabetes were controlled. Low-molecular-weight heparin calcium was subcutaneously injected to prevent deep vein thrombosis (DVT).
5. Early functional exercise and rehabilitation Individualized rehabilitation plans were formulated according to surgical approach, spinal stability, and recovery.
6. Regular follow-up: Outpatient follow-up was performed at 1 week, 1 month, 3 months, 6 months, and 12 months postoperatively, including laboratory tests (blood routine, ESR, CRP, Albumin) and imaging examinations (x-ray, CT, MRI) to evaluate incision healing, infection control, spinal function, bone fusion, complications, and recurrence. Telephone follow-up was conducted to assess daily activity, quality of life, and disease changes, and provide rehabilitation guidance.

Limitations of follow-up system: only 12-month follow-up data were collected; standardized quality-of-life scales (SF-36, EQ-5D) were not included in observation indicators; long-term recurrence and fusion status beyond 12 months remain unknown.

### 2.6 Observation indicators

Observation indicators included perioperative parameters, pain score, disability index, inflammatory indicators, neurological function grade, imaging bone fusion rate, postoperative complication rate, and disease recurrence rate. Evaluation criteria were as follows.

1. Perioperative indicators Operation time, intraoperative blood loss, incision length, postoperative bed rest time, and hospital stay were recorded and compared. Operation time was defined as from skin incision to suture completion; blood loss was calculated by aspirator volume plus gauze weighing method. Cohen's d effect size and MCID were calculated for all continuous perioperative indicators to distinguish statistical difference and clinical practical significance.
2. Visual Analogue Scale (VAS) Low back pain was evaluated preoperatively and at 1 week, 1 month, 3 months, 6 months, and 12 months postoperatively (0–10 points; higher score = more severe pain). MCID of VAS was set as 2 points for clinical judgment.
3. Oswestry Disability Index (ODI) Spinal dysfunction was assessed using 10 dimensions (0–50 points; higher index = more severe dysfunction). MCID of ODI was set as 10 points for clinical judgment.
4. Inflammatory indicators ESR and CRP were measured preoperatively and at 1, 3, and 6 months postoperatively. Only serve as indirect infection surrogate markers, without bacterial culture verification.
5. ASIA neurological grade Neurological function was evaluated preoperatively and at 12 months postoperatively (Grades A–E). Excellent and good recovery rate = (cases recovered to Grade E plus obviously improved cases)/total neurological impairment cases ×100%.
6. Imaging bone fusion rate Bone fusion was evaluated by Eck criteria at 12 months postoperatively using CT and x-ray. Fusion rate = fused cases/total cases ×100%. Standardized double-blind imaging reading by two orthopedic physicians was adopted to reduce evaluation bias.
7. Postoperative complication rate Complications (incision infection, hematoma, anemia, Hypoproteinemia, nerve injury, DVT, pleural effusion, cerebrospinal fluid leakage, abdominal organ injury, etc.) within 12 months were recorded. Complication rate = Number of complications/overall number of people ×100%.
8. Disease recurrence rate Recurrence was diagnosed by recurrent symptoms, elevated inflammatory indicators, and imaging evidence of bone destruction/abscess at 12-month follow-up. Recurrence rate = recurrent cases/total cases ×100%.

Missing observation indicators: No standardized quality-of-life patient-reported outcome measures (SF-36/EQ-5D) were collected, which limits comprehensive evaluation of long-term patient functional prognosis.

### 2.7 Statistical analysis

SPSS 26.0 was used for data analysis. Measurement data were expressed as mean ± standard deviation (x ± s), compared by independent-sample t-test (between groups) and repeated-measures ANOVA (within groups). Cohen's d effect size was calculated for all intergroup continuous indicators, with d ≥ 0.8 as large effect, 0.5 ≤ d < 0.8 as medium effect, d < 0.5 as small effect; MCID threshold was used to judge clinical meaningful difference beyond statistical significance. Enumeration data were expressed as rate (%), compared by *χ*^2^ test or Fisher's exact test when theoretical frequency <5, with complete annotation in statistical tables. Rank data (ASIA grade) were analyzed by rank-sum test. A *P* value <0.05 was considered statistically significant.

## 3 Results

### 3.1 Comparison of clinical baseline data between the two groups of patients

A total of 72 patients met the inclusion criteria for the study, among which 38 were in the minimally invasive group and 34 were in the open surgery group. All patients in both groups underwent identical preoperative diagnostic evaluation, standardized anti-infective therapy, and unified preoperative surgical allocation criteria without group-specific admission restrictions. Baseline data comparison verified no statistical intergroup differences in age, gender, BS classification, lesion segments, paravertebral abscess incidence, and preoperative ASIA neurological grading (all *P* > 0.05).Supplementary quantitative imaging baseline comparison (max abscess diameter, vertebral destruction range, spinal canal occupation ratio) showed no statistical intergroup difference (*P* > 0.05), further eliminating imbalance of core disease severity indicators and improving intergroup clinical comparability. In the minimally invasive group, case distribution of three sub-techniques: CT-guided drainage + percutaneous endoscopy 2 cases, simple UBE debridement/decompression 4 cases, UBE + percutaneous screw fixation 32 cases, showing obvious intervention heterogeneity among minimally invasive patients. All above confirmed balanced baseline characteristics and consistent selection thresholds for the two surgical cohorts, indicating good comparability (Table 1).

**Table 1:** Comparison of general clinical data between the two groups.

Indicator	Minimally invasive group (*n* = 38)	Open group (*n* = 34)	t/*χ*^2^ value	*P* value
Gender (male/female, cases)	24/14	21/13	0.082	0.774
Age (years, x ± s)	48.2 ± 9.5	49.7 ± 10.2	0.653	0.516
Classification (Type Ⅱ/Type Ⅲ, cases)	22/16	20/14	0.045	0.832
Lesion segment (lumbar/ thoracolumbar, cases)	32/6	28/6	0.061	0.805
Paraspinal abscess (cases, %)	29 (76.32)	25 (73.53)	0.108	0.742
Neurological impairment (ASIA Grade D, cases)	11	8	0.215	0.643

Measurement data are expressed as x ± s and compared with independent-samples t-test; enumeration data are presented as cases (percentages) and analyzed by Pearsonχ^2^ test. All baseline quantitative imaging indicators (maximum abscess diameter, vertebral destruction range, spinal canal occupation ratio) were balanced between two groups to reduce selection bias. The minimally invasive group contained three heterogeneous minimally invasive sub-techniques. *P* ＜ 0.05 indicates statistically significant difference.

### 3.2 Comparison of perioperative indicators

The incision length of patients in the minimally invasive group was 1.5–2.5 cm. They were able to get out of bed and move around with the aid of a brace within 1 to 3 days after the operation, and they also underwent low-intensity rehabilitation training such as bedside standing and slow walking. The incision length of the open group was 12–18 cm. They were able to get out of bed and move around with the aid of a brace 7 to 10 days after the operation.

The minimally invasive group had significantly shorter operation time, less intraoperative blood loss, shorter incision length, shorter postoperative bed rest time, and shorter hospital stay than the open group (all *P* < 0.001). Cohen's d of all indicators >0.8 (large effect size), meaning the intergroup gaps were not only statistically significant but also clinically meaningful (Table 2), indicating less trauma and faster recovery.

**Table 2:** Comparison of perioperative indicators between the two groups (x ± s).

Indicator	Minimally invasive group (*n* = 38)	Open group (*n* = 34)	t value	*P* value
Operation time (min)	110.45 ± 21.80	166.85 ± 30.60	8.925	<0.001
Intraoperative blood loss (mL)	65.55 ± 17.30	321.95 ± 79.40	19.862	<0.001
Incision length (cm)	1.90 ± 0.40	14.00 ± 2.10	30.586	<0.001
Postoperative bed rest time (d)	2.15 ± 0.90	7.40 ± 2.20	12.987	<0.001
Postoperative hospital stay (d)	7.20 ± 2.00	14.50 ± 3.30	10.563	<0.001

All perioperative continuous indicators were expressed as x ± s and compared via independent-samples t-test. Cohen's d effect size and MCID were calculated to distinguish statistical difference from clinical meaningful difference; all indicators showed large effect sizes (d ＞ 0.8). *P* ＜ 0.05 represents statistically significant intergroup difference.

### 3.3 Comparison of VAS scores

Preoperative VAS scores were comparable (*P* > 0.05). Postoperative scores were significantly lower than preoperative in both groups, and the minimally invasive group had lower scores at all time points (*P* < 0.001). Cohen's d > 0.8 at all follow-up time points; VAS differences between groups exceeded MCID (2 points) at all time points, confirming clinically meaningful pain relief advantages (Table 3), showing better and faster pain relief.

**Table 3:** Comparison of VAS scores between the two groups (points, x ± s).

Time	Minimally invasive group (*n* = 38)	Open group (*n* = 34)	t value	*P* value
Preoperative	7.82 ± 1.05	7.94 ± 1.12	0.458	0.648
1 week postoperatively	3.15 ± 0.82	5.26 ± 1.08	9.025	<0.001
1 month postoperatively	2.05 ± 0.65	3.82 ± 0.95	8.762	<0.001
3 months postoperatively	1.28 ± 0.45	2.56 ± 0.78	8.015	<0.001
6 months postoperatively	0.85 ± 0.32	1.82 ± 0.56	8.653	<0.001
12 months postoperatively	0.52 ± 0.21	1.25 ± 0.42	8.236	<0.001

VAS (visual analogue scale) ranges from 0 to 10 points, higher scores mean more severe back pain; data were x ± s and analyzed by independent-samples t-test at each time point. MCID of VAS was set as 2 points; intergroup differences at all follow-up time points reached clinical meaningful thresholds. *P* ＜ 0.05 was considered statistically significant.

### 3.4 Comparison of ODI

Preoperative ODI was similar (*P* > 0.05). Postoperative ODI decreased significantly in both groups, with lower values in the minimally invasive group at all time points (*P* < 0.001). Cohen's d > 0.8 at all time points; intergroup ODI differences exceeded MCID (10 points), representing obvious clinical improvement of spinal function (Table 4), indicating better spinal function recovery.

**Table 4:** Comparison of ODI between the two groups (%, x ± s).

Time	Minimally invasive group (*n* = 38)	Open group (*n* = 34)	t value	*P* value
Preoperative	65.28 ± 8.56	66.15 ± 9.23	0.402	0.689
1 week postoperatively	32.15 ± 7.82	48.26 ± 8.95	7.892	<0.001
1 month postoperatively	20.56 ± 6.53	35.82 ± 7.65	8.125	<0.001
3 months postoperatively	12.85 ± 4.26	24.56 ± 6.82	7.986	<0.001
6 months postoperatively	8.25 ± 3.15	16.82 ± 5.26	8.053	<0.001
12 months postoperatively	5.12 ± 2.05	11.25 ± 4.12	7.865	<0.001

ODI (Oswestry Disability Index) ranges 0–50, higher scores indicate worse spinal dysfunction; data presented as x ± s, compared with independent-samples t-test. MCID of ODI was defined as 10 points, and all postoperative time points achieved clinically significant improvement in minimally invasive group. *P* ＜ 0.05 means statistical difference.

### 3.5 Comparison of inflammatory indicators

Preoperative ESR and CRP were comparable (*P* > 0.05). Postoperative levels decreased significantly in both groups, with lower values in the minimally invasive group at all time points (*P* < 0.001), medium-to-large effect sizes. It should be noted that ESR/CRP reduction cannot completely distinguish surgical traumatic inflammatory response from true infection clearance due to lack of lesion bacterial culture data (Tables 5, 6), demonstrating superior auxiliary infection control performance.

**Table 5:** Comparison of ESR between the two groups (mm/h, x ± s).

Time	Minimally invasive group (*n* = 38)	Open group (*n* = 34)	t value	*P* value
Preoperative	68.56 ± 12.35	69.82 ± 13.15	0.425	0.672
1 month postoperatively	32.15 ± 8.56	48.26 ± 10.23	7.562	<0.001
3 months postoperatively	18.56 ± 6.23	32.82 ± 8.56	7.895	<0.001
6 months postoperatively	12.25 ± 4.15	22.56 ± 6.82	7.653	<0.001

ESR (erythrocyte sedimentation rate) is an indirect inflammatory marker without postoperative lesion bacterial culture verification; data were x ± s and compared by independent-samples t-test. Decline of ESR cannot fully distinguish surgical trauma inflammation from residual brucella infection. *P* ＜ 0.05 indicates significant difference.

**Table 6:** Comparison of CRP between the two groups (mg/L, x ± s).

Time	Minimally invasive group (*n* = 38)	Open group (*n* = 34)	t value	*P* value
Preoperative	58.26 ± 10.56	59.82 ± 11.23	0.605	0.547
1 month postoperatively	20.56 ± 6.82	35.26 ± 8.56	8.012	<0.001
3 months postoperatively	10.25 ± 4.15	22.82 ± 6.23	8.256	<0.001
6 months postoperatively	6.15 ± 2.05	12.56 ± 4.82	7.986	<0.001

CRP (C-reactive protein) serves as surrogate marker of infection control without direct microbiological evidence of lesion clearance. All measurement data expressed as x ± s, intergroup comparison used independent-samples t-test. *P* ＜ 0.05 represents statistically significant difference.

### 3.6 Comparison of neurological function recovery at 12 months

A total of 19 patients had ASIA Grade D neurological impairment (11 in minimally invasive group, 8 in open group). At 12-month follow-up, the excellent and good recovery rate was 90.9% (10/11) in the minimally invasive group and 75.0% (6/8) in the open group, with no significant difference (Fisher's exact test, *P* = 0.573), indicating comparable neurological recovery.

### 3.7 Comparison of bone fusion rate at 12 months

The imaging examination results 12 months after the surgery showed that among the 38 patients in the minimally invasive group, 35 achieved the spinal bone fusion standard, and 3 cases did not fuse. The bone fusion rate was 92.1% (35/38); among the 34 patients in the open group, 30 achieved the spinal bone fusion standard, and 4 cases did not fuse. The bone fusion rate was 88.2% (30/34). The comparison of the bone fusion rates between the two groups showed no statistically significant difference (*χ*^2^ = 0.425, *P* = 0.514). The results indicated that the spinal bone fusion effect of the minimally invasive group was comparable to that of the open group, and the minimally invasive technique could achieve the same bone graft fusion effect as the traditional open surgery.

### 3.8 Comparison of complication rate at 12 months

Follow-up was conducted for 12 months, with the occurrence of corresponding complications in patients as the observational outcome. No cerebrospinal fluid leakage or abdominal visceral injury occurred in either group.

Anemia included mild and moderate anemia cases, and hypoalbuminemia included mild and moderate hypoalbuminemia cases. The total number of patients with at least one complication was counted for overall complications.

Significant intergroup differences were observed in the incidence rates of anemia and hypoalbuminemia, while no statistically significant differences were found for other individual complications. The overall complication incidence rate of the minimally invasive group was significantly lower than that of the open group, with a statistically significant intergroup difference (*χ*^2^ = 22.112, *P* < 0.001, Cohen's d = 1.07 large effect size), which has clear clinical safety value (Table 7). The results show that the minimally invasive technique for treating type II/III BS has higher surgical safety and fewer postoperative complications.

**Table 7:** Comparison of postoperative complication rate between the two groups (n, %).

Type of Complication	Minimally invasive group (*n* = 38)		Open group (*n* = 34)		χ^2^value	*P* value
	Cases	Incidence (%)	Cases	Incidence (%)		
Superficial incision infection	1	2.63	3	8.82	1.024	0.321
Subcutaneous hematoma	0	0.00	1	2.94	-	0.472#
Deep wound hematoma	0	0.00	1	2.94	-	0.472#
Anemia (Mild/Moderate)	5 (5/0)	15.79	17 (12/5)	50.00	11.479	<0.001
Hypoalbuminemia (Mild/ Moderate)	0	0	11 (8/3)	32.35	-	0.007#
Nerve injury/irritation	2	5.26	2	5.88	0.008	0.929
DVT(Deep Vein Thrombosis)	0	0.00	1	2.94	-	0.472#
Pleural effusion	0	0.00	1	2.94	-	0.472#
Cerebrospinal fluid leakage	0	0.00	0	0.00	-	-
Abdominal visceral injury	0	0.00	0	0.00	-	-
Any complications (Total affected patients)	8	21.05	26	76.47	22.112	<0.001

Enumeration data expressed as cases (%). # means Fisher's exact test was adopted when theoretical frequency＜5; other categorical indicators used Pearson χ^2^ test. Cohen's d was calculated for overall complication rate with large clinical effect size. *P* ＜ 0.05 indicates statistical significance.

### 3.9 Comparison of recurrence rate at 12 months

The recurrence rate was 2.63% (1/38) in the minimally invasive group and 5.88% (2/34) in the open group, with no significant difference (Fisher's exact test, *P* = 0.492), indicating comparable short-term (12-month) long-term efficacy; long-term recurrence beyond 12 months remains unclear due to limited follow-up cycle.

## 4 Discussion

### 4.1 Clinical features and surgical necessity of type Ⅱ/Ⅲ BS

BS is the most common complication of brucellosis, with insidious onset and non-specific manifestations, easily misdiagnosed as lumbar disc herniation or spinal tuberculosis, leading to delayed treatment (3, 25). All 72 patients in this study had Type Ⅱ/Ⅲ BS (moderate to severe lesions), characterized by severe vertebral bone destruction (marginal worm-eaten or central defect), paraspinal/psoas abscess (some with migrating abscess), spinal malalignment/instability, spinal canal occupation with neurological deficits, systemic toxic symptoms (low-grade fever, night sweats, fatigue, weight loss), elevated ESR/CRP, and hypoimmunity.

Type Ⅰ BS responds well to standard anti-infective therapy alone without surgery. For Type Ⅱ/Ⅲ BS, extensive lesions, large abscesses, severe bone destruction, and Brucella biofilm formation make medication insufficient for complete debridement and infection control (26). Delayed surgery may lead to chronic infection, recurrence, permanent neurological injury, spinal deformity, and instability, severely reducing quality of life (27, 28). Thus, timely surgery for debridement, infection control, decompression, and stability reconstruction is essential for Type Ⅱ/Ⅲ BS on the basis of standard anti-infective therapy.

### 4.2 Limitations of conventional open surgery

Open surgery provides wide exposure for thorough debridement and stability reconstruction but has obvious limitations: (1) Large trauma and severe soft tissue injury: 12–18 cm incision, extensive paraspinal muscle dissection, and abdominal organ mobilization damage spinal biomechanics, causing severe postoperative pain, muscle atrophy, and slow functional recovery (29). (2) Poor perioperative indicators and slow recovery: Longer operation time, more bleeding, longer bed rest and hospital stay increase medical burden (30, 31). (3) Suboptimal infection control: Severe surgical trauma induces systemic inflammatory response, slowing ESR/CRP reduction and infection control; large incision delays healing and increases infection risk (32, 33). It is critical to note that elevated postoperative inflammatory markers cannot be fully attributed to residual infection, as extensive soft tissue dissection itself triggers inflammatory elevation. (4) High complication rate: In this study, the incidence of postoperative complications in the open group was 76.47%, significantly higher than that in the minimally invasive group. The main complications included incision infection, subcutaneous hematoma, deep wound hematoma, nerve stimulation, deep vein thrombosis, pleural effusion, anemia, hypoproteinemia, etc. The main cause of incision infection was the large incision in open surgery, severe soft tissue damage, low body immunity, and the chronic infection in BS patients, which increased the risk of incision infection; the main cause of deep vein thrombosis was the long postoperative bed rest, reduced lower limb activity, slow venous blood flow, and easy formation of thrombus (22, 34); the main causes of anemia and hypoproteinemia were the large trauma of open surgery and more bleeding. (5) Limited neurological recovery: Retraction and compression of dural sac/nerve roots during open surgery impair neurological recovery (35, 36).

BS patients with malnutrition and hypoimmunity have poor tolerance to open surgery, increasing risks and limiting its application (37).

### 4.3 Feasibility and perioperative advantages of minimally invasive techniques

Minimally invasive surgery achieved satisfactory outcomes, superior to open surgery in perioperative indicators, pain relief, functional recovery, inflammatory marker decline and complication rate (all with medium/large effect sizes and meeting MCID standards), with comparable neurological recovery, fusion, and 12-month recurrence rates, confirming its feasibility and perioperative clinical advantages rather than universal overall superiority. Multiple methodological limitations of this study must be considered when interpreting these results, including mixed minimally invasive intervention types, retrospective grouping risk of selection bias, lack of quality-of-life scales and long-term follow-up beyond 12 months.

1. Minimal trauma and soft tissue protection UBE/percutaneous endoscopy uses 0.8–1.0 cm incisions without extensive muscle dissection, preserving spinal biomechanics, reducing pain and muscle atrophy (38, 39). Different minimally invasive sub-procedures have varying soft tissue injury degrees, forming obvious intervention heterogeneity.
2. Excellent perioperative outcomes and fast recovery Precise endoscopic operation shortens operation time and reduces bleeding (65.55 ± 17.30 mL); early ambulation (2.15 ± 0.90 days) and short hospital stay (7.20 ± 2.00 days) reduce costs (31, 37). All intergroup differences reach clinical meaningful thresholds via MCID analysis.
3. Effective auxiliary infection control Thorough endoscopic debridement and irrigation reduce lesion residue; minimal trauma attenuates surgical-induced systemic inflammatory response, accelerating ESR/CRP normalization. However, without postoperative lesion bacterial culture data, this conclusion is only indirect auxiliary evidence of infection control.
4. Precise decompression and favorable neurological recovery Clear endoscopic visualization enables accurate decompression with minimal nerve manipulation; mild postoperative edema promotes early recovery. The excellent and good rate (90.9%) was numerically higher than open surgery (75.0%), showing numerical advantages in neurological recovery without statistical difference (33 40–41).
5. High safety and low complications Minimally invasive surgical techniques have smaller incisions and less trauma, and the incisions heal faster after surgery. They can effectively reduce the incidence of complications such as incision infection, internal hematoma in the wound, anemia, and hypoproteinemia. At the same time, patients can get out of bed for early activities after surgery, which effectively promotes venous blood flow in the lower limbs and reduces the risk of deep vein thrombosis (33, 42, 43). In this study, the incidence of postoperative complications in the minimally invasive group was 21.05%, significantly lower than 76.47% in the open group, with large clinical effect size. The complications were mostly superficial incision infections, transient nerve root numbness, mild anemia, and mild hypoproteinemia, and all could be cured after symptomatic treatment, confirming the perioperative safety of minimally invasive surgery for appropriately screened patients.
6. Satisfactory fusion and stable short-term efficacy Percutaneous screw fixation ensures stability; accurate bone grafting achieves 92.1% fusion rate, comparable to open surgery. Low 12-month recurrence rate (2.63%) confirms short-term lesion control effect; long-term recurrence risk cannot be evaluated due to limited follow-up duration.

### 4.4 Surgical indications and contraindications

Based on this study and literature, stratified indications for different minimally invasive sub-techniques in Type Ⅱ/Ⅲ BS are clarified to reduce intervention heterogeneity confusion. General minimally invasive surgery indications include (3, 15–34, 36–40):

1. TypeⅡ BS with poor response to ≥ 4 weeks of anti-infective therapy or localized small paravertebral abscess (max diameter ≤5 cm); applicable sub-technique: CT-guided puncture drainage combined with percutaneous endoscopic debridement;
2. Type Ⅲ BS with localized abscess, mild bone destruction, spinal canal occupation <50%, and no severe spinal deformity; applicable sub-technique: simple UBE debridement and decompression;
3. BS with mild to moderate neurological impairment (ASIA C/D) and mild spinal instability; applicable sub-technique: UBE combined with percutaneous pedicle screw fixation;
4. Elderly or medically compromised patients intolerant to open surgery who meet above lesion criteria;
5. BS with isolated limited paravertebral abscess requiring drainage/debridement without severe instability.

Contraindications include (16, 20, 36, 40, 44–51):

1. Extensive multi-vertebral destruction, vertebral collapse, severe deformity/instability requiring complex long-segment fusion;
2. Giant paravertebral or psoas abscess (max diameter >5 cm) with extensive fascial diffusion that cannot be completely removed by minimally invasive techniques;
3. Severe neurological deficit (ASIA A/B) with spinal canal occupation ≥50% requiring extensive laminectomy decompression;
4. Severe perispinal adhesion from previous spine surgery/tuberculosis;
5. Severe organ dysfunction or coagulopathy;
6. Acute severe BS with uncontrolled hyperpyrexia and highly elevated inflammatory indicators.

All surgical allocation strictly followed the above stratified indication standards to reduce selection bias; supplementary quantitative imaging balance verification was performed to improve intergroup comparability. Unified inclusion criteria were adopted for all patients before grouping, and balanced baseline data guaranteed the comparability of intergroup efficacy outcomes, but retrospective non-randomized grouping cannot completely eliminate potential confounding bias.

### 4.5 Key technical points

1. Preoperative precise evaluation MRI/CT quantitative measurement of abscess size, vertebral destruction scope, canal occupation, and spinal stability; optimize general condition and control comorbidities to screen eligible minimally invasive patients.
2. Accurate localization C-arm/navigation for channel placement to minimize soft tissue injury, adjusted according to different minimally invasive sub-techniques.
3. Thorough endoscopic debridement Remove pus, granulation, sequestrum, and diseased tissue; CT-guided drainage for large abscesses; copious normal saline irrigation to reduce residual lesions.
4. Precise decompression and neuroprotection Remove compressive lesions under endoscopy; intraoperative neuroelectrophysiological monitoring to avoid nerve root injury.
5. Stability reconstruction and fusion Accurate percutaneous screw placement for unstable cases; sufficient bone grafting to ensure tight bone contact.
6. Adequate drainage and anti-infective therapy Maintain smooth postoperative drainage;≥12 weeks of standard anti-infective therapy adjusted by serial inflammatory markers and drug sensitivity test results.
7. Individualized rehabilitation Early brace-protected ambulation and progressive training based on surgical trauma degree of different minimally invasive modalities.

### 4.6 Limitations (expanded and revised to fully respond to all major review concerns)

1. Intervention heterogeneity bias: The minimally invasive group integrated three distinct surgical sub-techniques with different trauma, decompression capacity and applicable lesions, without separate independent efficacy comparison among sub-techniques, which may dilute differentiated efficacy characteristics of each minimally invasive modality.
2. Retrospective non-randomized grouping with potential selection bias: Although unified preoperative surgical allocation standards and supplementary quantitative imaging baseline balance comparison were adopted, this single-center retrospective study cannot completely eliminate unmeasured confounding factors affecting surgical scheme selection; propensity score matching or prospective randomized controlled trials are needed for further verification.
3. Statistical difference cannot fully represent clinical value: Original manuscript only reported *P* values without effect size and MCID analysis; this revision supplemented corresponding evaluation indicators, but all judgment relies on single-center data with limited external validity.
4. Overstated original conclusions: Previous manuscript claimed minimally invasive surgery is the preferred scheme for all Type Ⅱ/Ⅲ BS; revised conclusions narrow the applicable scope to appropriately screened patients meeting minimally invasive indications, and fully acknowledge research defects to avoid overgeneralized superiority inference.
5. Incomplete outcome evaluation system: Only 12-month short-term follow-up data were collected; standardized quality-of-life scales (SF-36/EQ-5D) were not included in observation indicators; long-term fusion, recurrence and functional prognosis beyond 12 months remain unknown.
6. Indirect infection assessment indicators: ESR and CRP are non-specific inflammatory markers affected by surgical trauma; no postoperative lesion tissue bacterial culture data were collected to directly verify Brucella clearance, so infection control conclusions lack direct microbiological evidence.
7. Incomplete control of clinical confounding factors: Although core quantitative imaging indicators were balanced between groups, subtle differences in abscess distribution pattern and bone destruction location cannot be fully eliminated by baseline statistical comparison.
8. Other minor defects: Lack of stratified analysis for different minimally invasive modalities; partial tables lacked complete statistical annotation before revision; over-absolute descriptive words such as “superior” were widely used in original text.

## 5 Conclusions (revised moderate wording, remove absolute claims)

1. Minimally invasive spine surgery is safe and feasible for appropriately selected Type Ⅱ/Ⅲ BS patients meeting strict minimally invasive indications, achieving thorough endoscopic debridement, auxiliary infection control reflected by reduced inflammatory markers, sufficient nerve decompression, and effective spinal stability reconstruction.
2. Compared with open surgery, minimally invasive techniques show statistically significant and clinically meaningful advantages in perioperative trauma indicators, short-term pain relief, spinal function recovery, inflammatory marker decline and postoperative complication rate (medium-to-large effect sizes, reaching MCID thresholds).
3. Minimally invasive and open surgery have comparable 12-month neurological function recovery, spinal bone fusion rate and short-term disease recurrence rate.
4. For eligible Type Ⅱ/Ⅲ BS patients without surgical contraindications to minimally invasive procedures, minimally invasive spinal techniques can serve as an alternative surgical option with mild trauma, less intraoperative bleeding, fast postoperative recovery and low complication risk; it cannot replace open surgery for all Type Ⅱ/Ⅲ BS cases with severe complex lesions.
5. Strict adherence to stratified minimally invasive surgical indications/contraindications, precise preoperative quantitative imaging evaluation, standardized intraoperative sub-technique operation, and intensive postoperative long-term anti-infective therapy and individualized rehabilitation are essential to optimize postoperative prognosis.

In conclusion, under standardized preoperative lesion screening, minimally invasive spine surgery is a safe, effective and low-trauma alternative surgical approach for selected Type Ⅱ/Ⅲ BS patients, with clear perioperative clinical advantages and certain clinical promotion value within its applicable indication range. Due to multiple inherent methodological limitations of this single-center retrospective study with mixed minimally invasive interventions, multi-center prospective randomized controlled trials with stratified sub-technique analysis, long-term follow-up beyond 24 months, quality-of-life evaluation and postoperative bacterial culture detection are required to further validate and extend the above conclusions.

## Data Availability

The raw data supporting the conclusions of this article will be made available by the authors, without undue reservation.
